# Association between prenatal maternal infection and disordered eating behaviours in adolescence: a UK population-based prospective birth cohort study

**DOI:** 10.1017/S0033291719000795

**Published:** 2020-04

**Authors:** Francesca Solmi, Bianca L. De Stavola, Golam M. Khandaker, Cynthia M. Bulik, Christina Dalman, Glyn Lewis

**Affiliations:** 1 Division of Psychiatry, University College London, London, UK; 2 Great Ormond Street Institute of Child Health, UCL, London, UK; 3 University of Cambridge, Cambridge, UK; 4 Cambridgeshire and Peterborough NHS Foundation Trust, Cambridge, UK; 5 Department of Psychiatry, University of North Carolina at Chapel Hill, Chapel Hill, USA; 6 Department of Nutrition, University of North Carolina at Chapel Hill, Chapel Hill, USA; 7 Department of Medical Epidemiology and Biostatistics, Karolinska Institutet, Stockholm, Sweden

**Keywords:** ALSPAC, eating disorders, epidemiology, inflammation, prenatal infections

## Abstract

**Background:**

Prenatal infections have been proposed as a putative risk factor for a number of psychiatric outcomes across a continuum of severity. Evidence on eating disorders is scarce. We investigated whether exposure to prenatal maternal infections is associated with an increased risk of disordered eating and weight and shape concerns in adolescence in a large UK birth cohort.

**Methods:**

We used data from the Avon Longitudinal Study of Parents and Children. The primary exposure was maternal experience of infections at any time in pregnancy. Study outcomes were presence of any, monthly or weekly disordered eating at 14 and 16 years of age, and weight and shape concerns at 14 years. We defined the causal effect of the exposure on these outcomes using a counterfactual framework adjusting our analyses for a number of hypothesised confounders, and imputing missing confounder data using multiple imputation.

**Results:**

In total, 4884 children had complete exposure and outcome data at age 14 years, and 4124 at 16 years. Exposed children had a greater risk of reporting weekly disordered eating at both age 14 [risk difference (RD) 0.9%, 95% confidence interval (CI) −0.01 to 1.9, *p* = 0.08] and 16 (RD 2.3%, 95% CI 0.6–3.9, *p* < 0.01), though evidence of an association was weak at age 14 years. Exposed children also had greater weight and shape concerns at age 14 years (mean difference 0.15, 95% CI 0.05–0.26, *p* < 0.01).

**Conclusions:**

Exposure to prenatal maternal infection is associated with greater risk of disordered eating in adolescence. This association could be explained by *in utero* processes leading to impaired neurodevelopment or altered immunological profiles. Residual confounding cannot be excluded.

## Introduction

A growing body of literature suggests that exposure to prenatal infections might have a role in the aetiology of a number of psychiatric conditions (Flinkkilä *et al*., 2016). Prospective studies using large general population samples have shown that incidence of non-affective psychoses (Buka *et al*., 2001; Brown *et al*., 2004; Mortensen *et al*., 2007; Xiao *et al*., 2009; Brown and Derkits, 2010; Khandaker *et al*., 2013), bipolar disorder (Parboosing *et al*., 2013) and autism spectrum disorders (Atladóttir *et al*., 2010; Canetta *et al*., 2014; Lee *et al*., 2015; Hornig *et al*., 2018) is greater among individuals whose mother had an infection during pregnancy (Brown *et al*., 2004; Xiao *et al*., 2009; Parboosing *et al*., 2013). In comparison, the study of eating disorders has received remarkably little attention in the epidemiological literature (Favaro *et al*., 2011; Lydholm *et al*., 2018).

An association between exposure to prenatal infections and eating disorders is plausible for several reasons. Individuals with eating disorders often have co-morbid autistic traits (Gesi *et al*., 2017; Christensen *et al*., 2019), psychotic symptoms (Koyanagi *et al*., 2016; Solmi *et al*., 2018) and mood disorders (Hudson *et al*., 2007). All of these conditions have been shown to be more common among people exposed to infections *in utero* (Zammit *et al*., 2009; Smith *et al*., 2010; Lee *et al*., 2015; Simanek and Meier, 2015). Depression and psychosis are associated with dysregulated immunological profiles and, particularly, with increased inflammatory marker levels in peripheral blood (Upthegrove *et al*., 2014; Goldsmith *et al*., 2016). A meta-analysis of clinical studies has shown that similar immunological abnormalities are also observed in people with anorexia nervosa (Solmi *et al*., 2015*b*; Dalton *et al*., 2018). Previous studies have also shown that exposure to prenatal infections is associated with impaired neurodevelopmental traits [e.g. set-shifting (Khandaker *et al*., 2013), IQ (Benros *et al*., 2015; Khandaker *et al*., 2018)], which are also observed in individuals with eating disorders, particularly anorexia nervosa, in general population and clinical samples (Gillberg *et al*., 2007; Lang *et al*., 2015).

To the best of our knowledge, only two investigations have tested the hypothesis that maternal infections during gestation are associated with increased risk of eating disorder in the offspring (Favaro *et al*., 2011). One found that children whose gestational period coincided with peak epidemics of chickenpox and rubella had an increased risk of developing anorexia nervosa (Favaro *et al*., 2011). However, due to its ecological design, this study could not ascertain whether mothers had actually contracted any of these infections, therefore causal inferences cannot be drawn. Another recent study, using Danish register data, did not find an association between maternal infections during the prenatal period and greater risk of behavioural syndromes, i.e. including eating disorders, in the offspring after adjustment for multiple comparisons (Lydholm *et al*., 2018). However, the authors did not report eating disorder-specific estimates; hence, it is unclear if a specific association might exist.

To overcome some of the limitations of previous research, we investigated whether exposure to prenatal infections was associated with an increased risk of disordered eating behaviours (i.e. binge-eating, purging, dieting, fasting) and cognitions (i.e. weight and shape concerns) in adolescence using longitudinal data from a UK birth cohort sample. Disordered eating behaviours such as binge-eating, purging, fasting and dieting, and weight and shape concerns represent core trans-diagnostic features of eating disorder psychopathology (American Psychiatric Association, 2013). Although not everyone engaging in disordered eating transitions to a diagnosis, research suggests that those who do are at an increased risk of developing an eating disorder (Stice, 2016). As eating disorders are relatively uncommon (Smink *et al*., 2013), using behavioural and cognitive phenotypes, relying on the hypothesis that eating disorders occur on a continuum of severity (Dancyger and Garfinkel, 1995; Tylka and Subich, 1999), can aide aetiological research in general population samples (Miller *et al*., 2009). However, it is not yet known whether individuals with more severe eating disorder presentations represent aetiologically distinct entities from low-threshold behaviours more common in the general population. Therefore, to capture these putative differences, in this study, we tested our hypothesis using both broad and narrow definitions of disordered eating.

## Method

### Sample

We used data from the Avon Longitudinal Study of Parents and Children (ALSPAC), a general population cohort study of women and their offspring. A total of 14 451 women with an expected delivery date between 1 April 1991 and 31 December 1992 residing in the former region of Avon were recruited. The core ALSPAC sample consists of 14 062 live births, of whom 13 988 were alive at one year. More details on the ALSPAC cohort can be found in previous publications (Boyd *et al*., 2013; Fraser *et al*., 2013) and on the study website (http://www.bristol.ac.uk/alspac). This also contains details of all the data that are available through a fully searchable data dictionary available at: http://www.bristol.ac.uk/alspac/researchers/our-data/.

In this study, we included children in the core ALSPAC sample who had complete data on our primary exposure. In case of twins, we excluded the second born (twin ‘B’). Birth order could not be a confounder of the association between our exposure and outcome, because both twins would be exposed to infection and there is no evidence that twin birth order is associated with eating disorder risk (Holland *et al*., 1984). Ethical approval for the study was obtained from the ALSPAC Law and Ethics committee and the Local Research Ethics committees.

### Exposures

At 18 weeks of gestation, participating mothers were asked using questionnaire whether they had had influenza, thrush, rubella, genital herpes, urinary tract infections (UTIs) or any other infection in the first trimester of pregnancy or from the fourth month of pregnancy until the interview. Possible answers were: ‘no; in the first 3 months; from the fourth month until now; or at both time points’. At 32 weeks of gestation and at 8 weeks postpartum, women were also asked whether they had experienced the same conditions in the previous 3 months (i.e. from approximately the fourth month of gestation until the seventh month) and during the last 2 months of pregnancy, respectively. Since we could not identify which type of infection(s) would be included in ‘any other infections’, we defined our primary exposure as maternal self-reported any infection (influenza, thrush, rubella, genital herpes, UTI) anytime during pregnancy. In sensitivity analyses, we also re-ran all models with an alternative definition of the exposure using this additional information.

### Outcomes: disordered eating behaviours, weight and shape concerns

Our primary outcomes were presence of disordered eating behaviours (any, monthly and weekly), and weight and shape concerns at age 14 years. As sensitivity analyses, we explored disordered eating behaviours at age 16 years, when disordered eating was more common, although data were affected by greater proportions of missing data because of attrition.

#### Disordered eating behaviours

At age 14 and 16 years, children answered postal questionnaires including a battery of questions adapted from the Youth Risk Behaviour Survey questionnaire (Brener *et al*., 1995) investigating the presence of disordered eating behaviours in the previous 12 months.

We used data from both time points to test the robustness and consistency of the observed associations (if any). Moreover, the prevalence of disordered eating (and particularly of purging behaviours) increases from early to mid-adolescence in this sample. Therefore, although there is greater outcome missingness at age 16 years, the overall prevalence of the outcome is greater, increasing the statistical power of our analyses. Adolescents were asked whether they had fasted (i.e. not eaten in 24 h to lose weight), purged (i.e. thrown up or used laxative for weight loss) or binge eaten (i.e. eaten a large amount of food in a short period of time with a sense of loss of control) and with which frequency (never, less than monthly, monthly and weekly/daily). They were also asked if they had dieted and, if so, how frequently and for how long. As dieting is a very common behaviour, particularly among girls (Neumark-Sztainer *et al*., 2007), we only defined as having dieted adolescents who reported being on a diet ‘several times’, ‘often’ or ‘always’’ on a diet, and if they reported dieting for at least 1 month and up to 12 months continuously. This allowed us to restrict this definition to the more severe end of the dieting spectrum and align its definition with that of other behaviours we considered. These questions have been previously validated [questions on binge-eating were validated by Field *et al*. (2004)] and used in a number of previous studies (Micali *et al*., 2015*a*, 2017; Solmi *et al*., 2015*a*, 2018; Sonneville *et al*., 2015).

From these four behavioural variables, we created two outcome variables. First, an overall indicator denoting whether the adolescents experienced any dieting, binge-eating, purging or fasting on at least a monthly basis. Second, a variable indicating whether the adolescent had any monthly disordered eating behaviours and/or dieting; or any weekly disordered eating behaviours (bingeing, purging, fasting) and/or also dieted. We created this second variable to test the presence of threshold effects in relation to more severe disordered eating presentations, as weekly disordered eating episodes are the threshold defined by DSM-5 diagnoses. (More details are provided in online Supplementary Material.)

#### Weight and shape concerns

Weight and shape concerns were measured only at age 14 years with three questions from the McKnight Risk Factor Survey (Shisslak *et al*., 1999) asking ‘in the past year’ (1) ‘how happy have you been with the way your body looks?’; (2) ‘how much has your weight made a difference to how you feel about yourself?’; and (3) ‘how much have you worried about gaining a little weight (as little as 1 kilo)?’. Using a four-point Likert scale ranging from 0 (‘very happy’ for question 1, ‘not at all’ for questions 2 and 3), to 3 (‘very unhappy’ for question 1, ‘a lot’ for questions 2 and 3), we combined individual items to create a composite scale ranging from zero to nine, with higher scores indicating greater weight and shape concerns. This scale has good internal consistency given the low number of items (Chronbach's *α* = 0.66) and has previously been used in this sample (Micali *et al*., 2015*b*).

### Causal assumptions

**Fig. 1.**
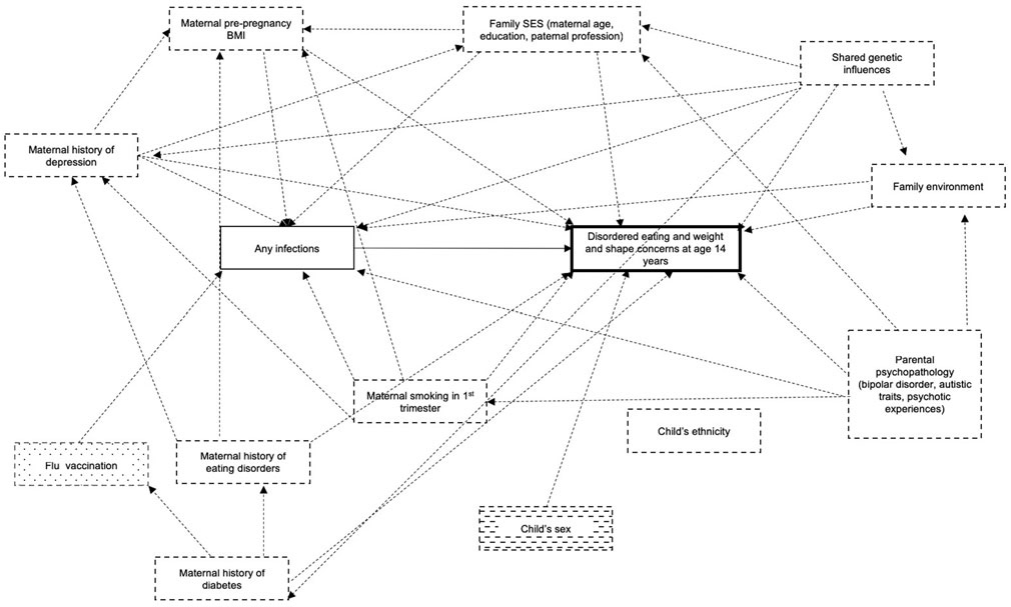
Direct Acyclic Graphs describing the causal assumptions for our analytical models.

In Fig. 1, we show our causal assumptions depicted using Direct Acyclic Graphs (DAGs) (Greenland *et al*., 1999) and the web programme DAGitty (Textor *et al*., 2011). Based on our assumptions, in order to estimate the total effect of the exposure (prenatal infections) on each of the outcomes (eating disorder behaviours and cognitions), it would be sufficient to adjust our models for: maternal depression, maternal pre-pregnancy body mass index (BMI), family socio-economic status (captured by: maternal age, paternal profession and maternal highest education), smoking in the first trimester of pregnancy and lifetime diabetes. We provide extensive details on the rationale for inclusion of each of these variables in online Supplementary Material.

### Confounders

Maternal depression, maternal pre-pregnancy BMI and lifetime history of diabetes were measured at 12 weeks of gestation. Women were asked whether they had ever suffered from severe depression. Possible answers were: no; yes, recently; yes, in the past, which we recoded as a binary variable (never/yes, at some point). Maternal pre-pregnancy BMI was calculated as kg/m^2^ from maternally-reported measures of height and pre-pregnancy weight and used as a continuous variable. History of diabetes was also self-reported (possible answers: no/yes).

At 18 weeks of gestation, women were asked how many cigarettes they had smoked per day in the first 3 months of pregnancy. Possible answers were none to over 30, which we recoded as a binary variable (none/one or more). At 32 weeks of gestation, women reported their highest academic education level (compulsory/non-compulsory) and the profession of their partner (manual/non-manual). Although these questions were asked after the exposure, it is unlikely that for a large proportion of the sample these would have changed since the beginning of the pregnancy, hence were used as potential confounders. Maternal age at delivery was derived from the date of delivery and the mother's date of birth collected at study enrolment.

### Statistical analyses

We described the sample characteristics using means and standard deviations and frequencies with proportions.

To investigate the association between exposure to prenatal infections (denoted *X*) and each of our outcomes (*Y*), we adopted a counterfactual framework approach to define the causal effect of *X* on *Y*, denoted hereafter average causal effect (ACE), as the difference of mean potential outcomes (POs) (Hernan and Robins, 2019). Let *Y^x^* represent the outcome that would occur had the exposure level be set to take the value *X* = *x*, and *E*(*Y^x^*) the PO averaged across the population of interest. For a binary exposure taking a value 0 or 1,
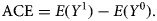


For binary outcomes, such as most of those studied here, the ACE is the comparison of two risks and therefore is a risk difference (RD) measure; when the outcome of interest is a count, it measures the difference in the mean number of events (MD). This definition has a causal connotation as it compares outcomes in worlds where all individuals in the populations are exposed *v.* when no one is exposed. Estimation was implemented in Stata 15 (StataCorp, 2017) with the *teffects ra* (i.e. treatment effects with regression adjustment) command, under the assumption of no unmeasured confounding (or equivalent, conditional exchangeability) and the technical assumptions of no interference and counterfactual consistency (Stata Press, 2017). This procedure fits separate regression models for the outcome on the previously identified confounders separately for each exposure level and averaging the outcomes predicted by each model over all the data to estimate *E*(*Y*^1^) and *E*(*Y*^0^). For binary outcomes, we used a logistic regression model to predict the POs; for count outcomes, we used Poisson regression. Results include the mean PO in the unexposed (to reflect the reference level) and, for binary outcomes, also the estimated causal risk ratios (reported as percentage risk increases). For results based on Poisson regression, the inference was based on robust standard errors to account for event over-dispersion. To investigate the impact of measured confounding, we also report the estimated crude associations between each exposure and each outcome.

We dealt with the effect of missing data on our estimates by using multiple imputation with chained equations, on the assumption that data would be missing at random, conditionally on the variables included in the models. We imputed 50 datasets using the Stata *mi impute chained* command using linear, logistic, multinomial logistic regression models according to the nature of the variable imputed. In our imputation model, we included all variables used in our analysis model and a list of auxiliary variables which we hypothesised would be (or there is evidence in the literature that are) associated with exposure, outcome and missingness. Here, we present the results based on participants with complete exposure and outcome and missing, and hence imputed, confounder data. In online Supplementary Material, we present the findings of complete case analyses. All analyses were conducted in Stata 15 (StataCorp, 2017).

## Results

### Sample

Of all children in the core sample alive at one year restricted to singletons and one twin (*N* = 13 793), 10 202 children (73.9%) had available exposure data across the three trimesters of pregnancy. Of these, 4785 (46.9%) had available outcome data at 14 years of age and 4111 (40.3%) at age 16 years.

Table 1 shows the demographic characteristics of children with complete exposure data and outcome data at 14 years. The majority of mothers had only completed compulsory education (*n* = 2571, 53.9%), had a partner working in a non-manual profession (*n* = 2893, 63.8%), did not smoke in the first trimester of pregnancy (*n* = 4026, 84.4%) and did not have a history of severe depression (*n* = 4426, 93.2%). Mean age of mothers in the sample was 29.3 years [standard deviation (s.d.) = 4.4] and mean pre-pregnancy BMI was 22.8 (s.d. = 3.7).

**Table 1.** Sample characteristics and their distributions among exposed children (complete exposure and outcome at age 14 years, *n* = 4785)

	Total^a^	Any infection (excluding ‘other’)^b^
	*N* (%)	*N* (%)
Infections	4785 (100.0)	2034 (42.5)
Child's sex		
Male	2133 (44.6)	895 (42.0)
Female	2652 (55.4)	1139 (43.0)
Maternal education		
Compulsory	2571 (53.9)	1127 (43.9)
Non-compulsory	2197 (46.1)	898 (40.9)
Paternal profession		
Manual	1643 (36.2)	760 (46.3)
Non-manual	2893 (63.8)	1162 (40.2)
Smoking		
No	4026 (84.4)	1660 (41.2)
Yes	747 (15.7)	368 (49.3)
Maternal history of severe depression		
No	4426 (93.2)	1832 (41.4)
Yes	324 (6.8)	189 (58.3)
Maternal history of diabetes		
No	4702 (99.2)	2005 (42.6)
Yes	38 (0.8)	13 (34.2)
	Total	Any infection (excluding ‘other’)
	Mean (s.d.)	Mean (s.d.)
Maternal age at delivery	29.3 (4.4)	29.1 (4.5)
Maternal BMI	22.8 (3.7)	22.9 (3.8)

a Column percentages showing distribution of the variable in the sample.

b Row percentages, showing proportions with the outcome in each level of the confounder.

s.d., standard deviation.

A total of 2034 women (42.5%) reported the occurrence of at least one of our target infections (i.e. not including ‘other’ infections) during the whole prenatal period (see Table 1). As expected, infections did not vary by child's sex; however, they were more common in mothers with lower education and who had a partner working in a manual occupation. A greater proportion of mothers with lifetime depression and who smoked reported having an infection, but fewer with a history of diabetes did so. On average, mothers who experienced an infection were slightly younger, but had similar BMI compared with those who did not. When including ‘other’ infections, the proportion of mothers reporting any infection increased to 51.3% (*n* = 2453, see online Supplementary Table S1). Although mothers with depression and who smoked still reported more infections using this definition, now the latter were more common among mothers who were more educated.

#### Missing data

Among children with complete exposure data in the first trimester, outcome data were missing for 53.1% of children (*n* = 5417) at age 14 years, and for 59.7% (*n* = 6091) at age 16 years. Children who were male, with mothers who had lower education, were younger, smoked in pregnancy, had a history of depression, as well as those whose father worked in a manual profession were more likely to have missing outcome data at both time points. Exposure to prenatal infections was also associated with missing outcome data (see online Supplementary Table S2).

### Prevalence of disordered eating behaviours in adolescence

Of those with available exposure data, at age 14 years and age 16 years, 380 (7.9%) and 647 (12.8%) adolescents reported disordered eating behaviours, respectively. At age 14 years, 136 (2.8%) of the sample and at 16 years 241 adolescents (4.8%) reported that these behaviours occurred weekly. More detailed information on the prevalence of individual disordered eating behaviours is provided in online Supplementary Table S3.

### Exposure to prenatal infections and outcomes at 14 years of age

As shown in Table 2, children have a 7.4% estimated baseline risk in the absence of exposure [i.e. *E*(*Y*^0^)] of reporting any disordered eating [95% confidence intervals (CI) 6.4–8.3]; a 5.0% risk of reporting monthly behaviours (95% CI 4.2–5.9); and a 2.5% risk of reporting weekly behaviours (95% CI 1.9–3.2).

**Table 2.** Average causal effects of exposure to infections in pregnancy and study outcomes

	Crude association^a^ (95% CI), *p* value	Average causal effect^a^ (95% CI), *p* value	Potential outcome^b^ mean in unexposed	% Risk increase in exposed
Disordered eating 14 years (*N* = 4785)				
Any	0.017 (0.001–0.032), *p* = 0.0378	0.011 (−0.004 to 0.027), *p* = 0.1579	0.074 (0.064–0.083)	
Monthly	0.006 (−0.007 to 0.019), *p* = 0.3572	0.003 (−0.009 to 0.016), *p* = 0.6349	0.050 (0.042–0.059)	
Weekly	0.012 (0.002–0.022), *p* = 0.0220	0.009 (−0.001 to 0.019), *p* = 0.0802	0.025 (0.019–0.032)	**36.0**
Weight and shape concerns	0.183 (0.077–0.288), *p* = 0.0007	0.156 (0.050–0.262), *p* = 0.0039	2.261 (2.193–2.329)	**6.8**
Disordered eating 16 years (*N* = 4111)				
Any	0.038 (0.015–0.061), *p* = 0.0012	0.030 (0.007–0.053), *p* = 0.0101	0.145 (0.131–0.198)	**20.7**
Monthly	0.016 (−0.003 to 0.036), *p* = 0.1035	0.013 (−0.006 to 0.033), *p* = 0.1992	0.099 (0.087–0.111)	
Weekly	0.029 (0.012–0.045), *p* = 0.0007	0.023 (0.006–0.039), *p* = 0.0068	0.055 (0.046–0.065)	**41.8**

Samples based on participants with complete exposure and outcome available and thus vary across models.

**Confounders accounted for in ACE estimation**: maternal: age, education, lifetime history of diabetes and depression, pre-pregnancy BMI, smoking in the first trimester; and paternal profession.

a For disordered eating, these figures represent risk differences (we report these as proportions in the results section). For weight and shape concerns, these are mean differences.

b This potential outcome mean (POM) figure refers to the baseline risk of the outcome among the unexposed and, as such, should be interpreted as a proportion (i.e. multiplied by 100), which is how we present these figures in the Results section of manuscript. For weight and shape concerns, this represents a mean score instead.

There was some evidence that prenatal infection was associated with frequent (weekly) disordered eating behaviours in both unadjusted (difference in observed prevalence 1.2%, 95% CI 0.2–2.2, *p* < 0.01) and confounder-adjusted analyses (RD 0.9%, 95% CI −0.1 to 1.9, *p* = 0.08). The latter is equivalent to a 36% increase in risk. Less frequent disordered eating behaviour (monthly) or any disordered eating was not associated with prenatal infection (Table 2).

Average weight and shape concerns score in the unexposed group was 2.3 (95% CI 2.2–2.3). The observed mean difference (MD) in scores in the exposed group was (MD 0.18, 95% CI 0.07–0.29, *p* < 0.01). This estimate decreased in the confounder-adjusted model (MD 0.16, 95% CI 0.05–0.26, *p* ⩽ 0.01) representing a 6.8% increase in total weight and shape concerns score in the exposed group.

### Exposure to prenatal infections and outcomes at 16 years of age

Similar to what we observed at age 14 years, prenatal infection was associated with frequent (weekly) disordered eating behaviour at age 16 years, though evidence of an association was stronger at this age.

Table 2 shows that unexposed children have a 14.5% estimated baseline risk of reporting any disordered eating (95% CI 13.1–19.8); a 9.9% risk of reporting monthly behaviours (95% CI 8.7–11.1); and a 5.5% risk of reporting weekly behaviours (95% CI 4.6–6.5).

The observed difference in prevalence of any eating disorder behaviours was of 3.8% (unadjusted analysis; 95% CI 1.5–6.1). The causal RD for any disordered eating estimated accounting for the pre-defined confounders was smaller (RD 3.0%, 95% CI 0.7–5.3, *p* < 0.01). Prenatal infection was strongly associated with frequent (weekly) disordered eating behaviours in both unadjusted (difference in observed prevalence 2.9%, 95% CI 1.2– 4.5, *p* < 0.01) and confounder-adjusted analyses (RD 2.3%, 95% CI 0.6–3.9, *p* < 0.01). The latter is equivalent to a 41.8% increase in risk. We did not find an association between prenatal infection and less frequent disordered eating behaviour (monthly) (Table 2).

### Sensitivity and complete case analyses

Results of our analyses based on complete cases (see online Supplementary Table S4) were comparable to those from imputed models in terms of magnitude of the association, although here evidence of an association was stronger. When we included ‘other’ infections as part of our exposure (see online Supplementary Table S5), we also observed qualitatively similar associations, although there was weaker evidence of an association between prenatal infections and weekly disordered eating at 16 years. These findings were also comparable to those based on complete cases (see online Supplementary Table S6).

## Discussion

In this study, we found that children who were exposed prenatally to infections had increased risk of reporting weekly disordered eating behaviours. Evidence for this association was stronger at age 16 years, possibly due to greater statistical power at this time point since the risk ratios (i.e. the proportion of increase in risk) were comparable at age 14 years (41.8% at age 16 years *v.* 36.0% at age 14 years, Table 2). Exposed children also had greater weight and shape concerns – a trans-diagnostic cognitive marker of eating disorders – at age 14 years.

Our findings of an increased risk of more frequent disordered eating behaviours in children exposed to prenatal infections are broadly in line with those of previous studies (Favaro *et al*., 2011; Lydholm *et al*., 2018).

### Strengths and limitations

To the best of our knowledge, this is the first study to investigate the association between exposure to prenatal infections and self-reported disordered eating in the general population. In this study, we relied on data from the ALSPAC dataset, a large birth cohort covering a wealth of biological and environmental information on children and their families. In building our analytical models – rooted in a counterfactual framework approach – we relied on pre-specified causal assumptions, outlined using DAGs, which allowed to establish the minimum sets of confounders necessary to calculate a causal effect (if our assumptions are correct and there is no residual confounding). Our exposure was prospectively collected at assessments that took place throughout pregnancy, thus minimising recall bias.

Data on disordered eating behaviours were self-reported by adolescents, which represents an advantage over parental report, as there is evidence that parents might miss key behaviours such as binge eating and purging (Swanson *et al*., 2014). This is particularly important as adolescents who binge and purge are also often undetected in clinical settings, making general population studies key when investigating these behaviours. Our disordered eating definitions have not been validated against clinical diagnoses and thus their diagnostic validity could have been improved from combination with clinical interviews.

To study more ‘severe’ presentations, we created frequency cut-offs modelled after DSM-5 criteria for eating disorders. Previous studies have also found that these behaviours –whose prevalence is higher than that of diagnoses in the general population – are associated with substantial comorbidity (Micali *et al*., 2015*a*; Solmi *et al*., 2015*a*; Micali *et al*., 2017; Solmi *et al*., 2018) making the study of their aetiology an important aim in itself. Furthermore, the use of trans-diagnostic definitions of behaviours and cognitions in general population samples has the advantage of capturing presentations that might index increased susceptibility to develop threshold diagnoses and reduce limitations (e.g. low power and selection bias) arising from the use of clinical diagnoses.

A number of limitations should also be acknowledged. First, infections were self-reported by the mother and this could result in some measurement error. In order to minimise this error and to increase statistical power, we pooled all infections together across the three trimesters. Because our outcome is relatively uncommon, we also lacked statistical power to investigate associations by type of infection, particularly as some of these were rather rare due to widespread immunisation (e.g. rubella), or by timing of infections. We were also unable to investigate the presence of dose–response associations (e.g. number of infections in pregnancy) as for some women some of the questionnaires might have referred to the same time period, and hence, we could not exclude double counting.

Literature suggests an association between Type I diabetes and eating disorders (Raevuori *et al*., 2014; Zerwas *et al*., 2017; Hedman *et al*., 2018). Although we adjusted our analyses for maternal lifetime history of diabetes, we did not have information on type of diabetes, and hence, this could have resulted in some measurement error.

ALSPAC is affected by high levels of attrition. We used multiple imputation to impute missing confounder data; however, the amount of missingness (>50%) in the outcome was too large for confident imputation in the absence of established literature from longitudinal studies of antecedents of eating disorders. Indeed, imputing outcomes under these circumstances is discouraged (White *et al*., 2011). We could not control our analyses for a number of hypothesised confounders, such as genetic predisposition, family environment and parental psychopathology relevant to exposure and outcome. Future investigations should aim to triangulate these findings using study designs which can better account for these largely unobservable factors.

### Interpretation of findings

Several hypotheses have been advanced to explain the observed excess of mental health problems in individuals exposed to infections prenatally. One possibility is that exposure to maternal immune activation could directly affect foetal neurodevelopment. Animal studies have reported that, in mice, exposure to prenatal infection results in reduced sociability (Malkova *et al*., 2012; Weber-Stadlbauer *et al*., 2017) and increased repetitive behaviours (Malkova *et al*., 2012). Deficits in social communication, elevated autistic traits and social anxiety are common in individuals with eating disorders (Christensen *et al*., 2019). Another hypothesis is that exposure to prenatal infection could predispose the offspring to autoimmune disorders. A number of studies have now shown that individuals with autoimmune disorders have a greater risk of eating disorders (Raevuori *et al*., 2014; Zerwas *et al*., 2017; Hedman *et al*., 2018). Finally, exposure to prenatal infections might be associated with alterations of the Hypothalamic Pituitary Adrenal (HPA) axis – involved in cortisol release and stress response – which might also be involved in the onset and maintenance of eating disorders (Lo Sauro *et al*., 2008). Future general population-based and experimental studies should attempt to test these potential mechanisms.

Our findings could also be explained by non-causal mechanisms, for instance residual confounding. Although we did not observe large attenuations in size or strength of the association once including indicators of socio-economic status (suggesting that the latter might not be an important confounder), we cannot exclude that these associations might be explained by residual genetic confounding or familial characteristics which we were not able to capture in this dataset. Future studies should attempt to control for these factors by using study designs that control for these unobserved factors and by triangulating different approaches.

In conclusion, we present novel findings of an association between exposure to prenatal infections and disordered eating presentations in adolescence. Our findings add to the existing evidence base linking prenatal exposure to infections to a range of psychopathological outcomes in the offspring, by showing that this effect might be relevant in the aetiology of eating disorders as well. Future studies should aim to investigate putative mechanisms and replicate these results in other settings and datasets possibly triangulating findings from different designs in order to strengthen causal inference.

## Supplementary material

For supplementary material accompanying this paper visit http://dx.doi.org/10.1017/S0033291719000795. click here to view supplementary material
