# Supplementary material for: Association between prenatal maternal infection and disordered eating behaviours in adolescence: a UK population-based prospective birth cohort study
Source: Psychol Med. 2019 May 2;50(6):927–35. doi: 10.1017/S0033291719000795 (PMC7191780; doi:10.1017/S0033291719000795)
Supplement: Supplementary file 1 [file S0033291719000795sup.zip › S0033291719000795sup001.docx]

**SUPPLEMENTAL MATERIAL**

Disordered eating behaviours

Adolescents were asked whether they had ever fasted for at least 24 hours to lose weight, and if they had used laxative or vomited for the same reason. Possible answers were: ‘no’, ‘less than once a month’, ‘one to three times per month’, ‘once a week’, ‘two to six times per week’. We considered as having ever fasted or purged, adolescents who reported engaging in these behaviours at least once a month (‘one to three times per month’ or more frequently’). Those who reported these behaviours less frequently than monthly were not considered as having disordered eating behaviours, as we could not estimate how frequently these behaviours would have occurred and wanted to minimise misclassification error. Adolescents were also asked how frequently they had eaten a large amount of food in a short period of time. Possible answers were: ‘never’, ‘less than once a month’, ‘one to three times per month’, ‘once a week’, ‘more than once per week’. Those who reported any episodes of overeating (i.e., regardless of frequency) were also asked whether, during those episodes, they had experienced a sense of loss of control, such as that they could not stop even if they had wanted to. Possible answers were: ‘no’, ‘sometimes’, or ‘usually’. We considered a child as having binge eaten if they had reported at least monthly episodes of overeating and if they had said that they ‘sometimes’ or ‘usually’ felt out of control during these episodes. From these three variables (i.e., fasting, purging, and binge eating), we also created additional ones indicating whether these disordered eating behaviours occurred: ‘never’, ‘monthly, but less than weekly’, or ‘weekly’. Finally, adolescents were asked whether they had dieted to lose weight. As dieting is a very common behaviour – particularly among girls – we only defined as having dieted ‘problematically’ adolescents who reported being several times’, ‘often’, or ‘always’ on a diet, and if they reported dieting for at least one month and up to 12 months continuously.

From these variable we created two outcome measures:

1. Any disordered eating: 0 = none, 1= binge eating, purging, fasting occurring at least monthly or any dieting.
2. Frequency of disordered eating: 0 = none/never; 1= monthly binge eating and/or monthly purging and/or monthly fasting and/or dieting; 2= binge eating, purging, or fasting occurring at least weekly plus dieting.

Causal assumptions

Maternal pre-pregnancy BMI has been associated with child disordered eating (1) and BMI is generally linked with an increased risk of infections(2). Maternal history of depression is associated with greater risk of eating disorders in the offspring(3) and evidence shows that individuals with depression have higher rates of infections and greater inflammation.(4) We hypothesised that familial socio-economic status (SES) could increase risk for infections (for instance via household crowding) or affect reporting due to better health literacy associated with higher levels of education. A recent study also found that lower SES is associated with greater disordered eating. (5) Maternal smoking has been associated with immunodeficiency (which would increase a person’s risk of contracting an infection), and we hypothesised it would be associated with disordered eating (in prep), potentially via alteration of the HPA axis or neurodevelopment. Recently, a number of studies have shown that individuals with eating disorders are more likely to have an autoimmune disease.(6, 7) Although we did not have information on type of diabetes, mothers were asked whether they had a history of diabetes. We included this variable as a confounder because flu (one of the most prevalent infections in our sample) vaccination is recommended for individuals with diabetes, hence we hypothesised mothers with diabetes could have different patterns of infections. In the absence of information on vaccination status, based on our assumptions, adjustment for diabetes status was sufficient.

Child’s sex was not included as a confounder as – although associated with the outcome – it could not have affected the exposure. We hypothesised that child’s ethnic group would not be associated with disordered eating patterns or with the exposure. We also hypothesised that genetic factors, parental mental health history (e.g. psychotic symptoms, bipolar disorder, autistic traits), and family environment could be associated with increased susceptibility of infection and psychopathology in adolescence. However, we could not adjust for these factors in the current analyses and we could not design a study that could account for them (e.g. twin/sibling comparisons).

Multiple imputation

Variables included in multiple imputation model: child’s sex; ethnicity; IQ at age 8 years; peer victimisation at age 8 years; mental health difficulties at age 7 years; self-esteem at age 8 years; sexual orientation at age 15 years; self-harm at age 16 years; leptin at age 9 years; BMI at age 7, 9, 11, 14, 16, and 18 years; emotional and restrained eating at age 14 years; psychotic symptoms at age 12 years; depressive symptoms at age 12 and 16 years; autistic traits at age 7, 11, 14, and 16 years; disordered eating at age 14 and 16 (as categorical variables). Maternal lifetime depression; pre-pregnancy BMI; infections in pregnancy; flu, urinary tract infections, or thrush in 1^st^, 2^nd^, and 3^rd^ trimester; education; smoking in 1^st^ trimester of pregnancy; lifetime history of eating disorders and diabetes; social class. Paternal social class.

**Supplemental table 1: Distribution of confounders across secondary exposure**

|  | **Total ^a^** | **Any infection**  **(including ‘other’) ^b^** |
| --- | --- | --- |
|  | **N (%)** | **N (%)** |
| **Proportion of infections** | 4,785 (100.0) | 2,453 (51.3) |
| **Child’s sex** |  |  |
| *Male* | 2,133 (44.6) | 1,094 (51.3) |
| *Female* | 2,652 (55.4) | 1,359 (51.2) |
| **Maternal Education** |  |  |
| *Compulsory* | 2,571 (53.9) | 1,283 (49.9) |
| *Non-compulsory* | 2,197 (46.1) | 1,161 (52.8) |
| **Paternal profession** |  |  |
| *Manual* | 1,643 (36.2) | 854 (52.0) |
| *Non-manual* | 2,893 (63.8) | 1,471 (50.9) |
| **Smoking** |  |  |
| *No* | 4,026 (84.4) | 2,031 (50.5) |
| *Yes* | 747 (15.7) | 415 (55.6) |
| **Maternal history of severe depression** |  |  |
| *No* | 4,426 (93.2) | 2,220 (50.2) |
| *Yes* | 324 (6.8) | 216 (66.7) |
| **Maternal history of diabetes** |  |  |
| *No* | 4,702 (99.2) | 2,415 (51.4) |
| *Yes* | 38 (0.8) | 17 (44.7) |
|  | **Total** | **Any infection**  **(including ‘other’)** |
|  | **Mean (SD)** | **Mean (SD)** |
| **Maternal Age at delivery** | 29.3 (4.4) | 29.4 (4.5) |
| **Maternal BMI** | 22.8 (3.7) | 22.9 (3.7) |

**Supplemental Table 2: Predictors of missing outcome data at 14 and 16 year assessments among participants with complete exposure (n = 10,202)**

|  | **Missing outcome** | |
| --- | --- | --- |
|  | **Age 14**  **(n = 5,417, 53.1%)**  **OR (95% CI)** | **Age 16**  **(n = 6,091, 59.7%)**  **OR (95% CI)** |
| Any infection (other excluded) | 1.25 (1.16, 1.35),  p=0.0119 | 1.27 (1.17, 1.37), p<0.0001 |
| Any infection (other included) | 1.11 (1.02, 1.20), p<0.0001 | 1.13 (1.04, 1.22), p=0.0032 |
| Child sex (female vs male) | 0.59 (0.55, 0.64), p<0.0001 | 0.52 (0.48, 0.56), p<0.0001 |
| Maternal education (non-compulsory vs compulsory) | 0.48 (0.44, 0.52), p<0.0001 | 0.43 (0.40, 0.47), p<0.0001 |
| Paternal profession (non-manual vs. manual) | 0.59 (0.55, 0.65), p<0.0001 | 0.52 (0.47, 0.56), p<0.0001 |
| Maternal depression (yes vs no) | 1.50 (1.30, 1.73), p<0.0001 | 1.69 (1.45, 1.98), p<0.0001 |
| Maternal diabetes (yes vs no) | 1.22 (0.79, 1.86), p=0.3614 | 1.36 (0.87, 2.11), p=0.1744 |
| Maternal Age | 0.93 (0.92, 0.94), p<0.0001 | 0.93 (0.92, 0.94), p<0.0001 |
| Smoked in 1^st^ trimester (yes vs no) | 1.96 (1.78, 2.17), p<0.0001 | 1.96 (1.77, 2.18), p<0.0001 |
| Maternal pre-pregnancy BMI | 1.02 (1.01, 1.03), p<0.0001 | 1.03 (1.02, 1.04), p<0.0001 |

**Supplemental Table 3: Prevalence of disordered eating behaviours at age 14 years and 16 years among those with complete exposure data**

| **Disordered eating behaviour** | **Age 14 years,**  **N (%)** | **Age 16 years,**  **N (%)** |
| --- | --- | --- |
| **Total** | **4,785** | **4,111** |
| Any disordered eating | 380 (7.92%) | 647 (12.78%) |
| Monthly disordered eating | 244 (5.10%) | 406 (8.03%) |
| Weekly disordered eating | 136 (2.84%) | 241 (4.75%) |
| Dieting | 194 (4.05%) | 272 (6.62%) |
| Fasting | 124 (2.59%) | 243 (5.91%) |
| Binge eating | 109 (2.28%) | 242 (5.89%) |
| Purging | 30 (0.63%) | 156 (3.79%) |

**Supplemental table 4: Association between any prenatal infections (excluding ‘others’) in pregnancy with disordered eating behaviours and cognitions (complete cases)**

|  | **Crude association^a^**  **(95% CI), p-value** | **Average Causal Effect  ^a^**  **(95% CI), p-value** | **Potential Outcome ^b^ Mean in unexposed** | **% risk increase in exposed** |
| --- | --- | --- | --- | --- |
| **Disordered eating 14 years**  **(N= 4,283)** |  |  |  |  |
| *Any* | **0.018 (0.002, 0.034), p=0.0264** | **0.013 (-0.002, 0.029), p=0.0989** | 0.067 (0.057, 0.077) | 19.4% |
| *Monthly* | 0.006 (-0.008, 0.020), p=0.3870 | 0.003 (-0.010, 0.017), p=0.6630 | 0.049 (0.040, 0.058) |  |
| *Weekly* | **0.014 (0.004, 0.024), p=0.0070** | **0.012 (0.002, 0.022), p=0.0212** | 0.019 (0.014, 0.025) | **59.8%** |
| *Weight and shape concerns* | **0.017 (0.054, 0.277), p=0.0038** | **0.142 (0.030, 0.254), p=0.0129** | 2.248 (2.175, 2.320) | **6.3%** |
| **Disordered eating 16 years**  **(N =3,670)** |  |  |  |  |
| *Any* | **0.037 (0.013, 0.061), p=0.0023** | **0.031 (0.006, 0.055), p=0.0128** | 0.142 (0.127, 0.157) | **21.4%** |
| *Monthly* | 0.015 (-0.006, 0.035), p=0.1642 | **0.011 (-0.010, 0.032), p=0.2897** | 0.098 (0.085, 0.111) |  |
| *Weekly* | **0.030 (0.012, 0.047), p=0.0008** | **0.025 (0.008, 0.043), p=0.0042** | 0.053 (0.043, 0.063) | **47.7%** |

**Multivariable model adjusted for**: maternal age, education, paternal profession, maternal depressive symptoms, maternal pre-pregnancy BMI, and smoking in the first pregnancy trimester.

**^a^** For disordered eating, these figures represent risk differences (we report these as proportions in the results section). For weight and shape concerns, these are mean differences.

**^b^** This potential outcome mean (POM) figure refers to the baseline risk of the outcome among the unexposed and, as such, should be interpreted as a proportion (i.e., multiplied by 100), which is how we present these figures in the Results section of manuscript. For weight and shape concerns, this represents a mean score instead.

**Supplemental table 5: Association between any prenatal infections (including ‘others’) with disordered eating behaviours and cognitions (complete exposure and outcome, imputed missing confounders)**

|  | **Crude association^a^**  **(95% CI), p-value** | **Average Causal Effect  ^a^**  **(95% CI), p-value** | **Potential Outcome ^b^ Mean in unexposed** | **% risk increase in exposed** |
| --- | --- | --- | --- | --- |
| **Disordered eating 14 years**  **(N= 4,844)** |  |  |  |  |
| *Any* | 0.013 (-0.002, 0.028), p=0.1033 | 0.010 (-0.005, 0.025), p=0.1892 | 0.073 (0.063, 0.084) |  |
| *Monthly* | 0.003 (-0.009, 0.016), p=0.6394 | 0.001 (-0.011, 0.014), p=0.8277 | 0.051 (0.042, 0.061) |  |
| *Weekly* | 0.011 (0.001, 0.021), p=0.0304 | 0.009 (0.001, 0.019), p=0.0484 | 0.024 (0.018, 0.031) | 37.5% |
| *Weight and shape concerns* | 0.120 (0.027, 0.212), p=0.0120 | 0.119 (0.014, 0.222), p=0.0257 | 2.265 (2.190, 2.339) | 5.2% |
| **Disordered eating 16 years**  **(N =4,111)** |  |  |  |  |
| *Any* | **0.034 (0.012, 0.056), p=0.0025** | **0.029 (0.008, 0.052), p=0.0083** | 0.141 (0.126, 0.157) | **20.6%** |
| *Monthly* | 0.019 (-0.001, 0.038), p=0.0503 | 0.017 (-0.002, 0.037), p=0.0831 | 0.096 (0.082, 0.109) | **17.7%** |
| *Weekly* | **0.021 (0.005, 0.037), p=0.0088** | 0.018 (0.002, 0.034), p=0.0246 | 0.056 (0.045, 0.066) | **32.1%** |

**Multivariable model adjusted for**: maternal age, education, paternal profession, maternal depressive symptoms, maternal pre-pregnancy BMI, and smoking in the first pregnancy trimester.

**^a^** For disordered eating, these figures represent risk differences (we report these as proportions in the results section). For weight and shape concerns, these are mean differences.

**^b^** This potential outcome mean (POM) figure refers to the baseline risk of the outcome among the unexposed and, as such, should be interpreted as a proportion (i.e., multiplied by 100), which is how we present these figures in the Results section of manuscript. For weight and shape concerns, this represents a mean score instead.

**Supplemental table 6: Association between any prenatal infections (including ‘others’) in pregnancy with disordered eating behaviours and cognitions (complete cases)**

|  | **Crude association^a^**  **(95% CI), p-value** | **Average Causal Effect  ^a^**  **(95% CI), p-value** | **Potential Outcome ^b^ Mean in unexposed** | **% risk increase in exposed** |
| --- | --- | --- | --- | --- |
| **Disordered eating 14 years**  **(N= 4,283)** |  |  |  |  |
| *Any* | **0.017 (0.001, 0.033), p=0.0345** | **0.014 (-0.001, 0.030), p=0.0726** | 0.065 (0.055, 0.076) | 21.8% |
| *Monthly* | 0.005 (-0.009, 0.018), p=0.4749 | 0.003 (-0.010, 0.016), p=0.6511 | 0.049 (0.040, 0.059) |  |
| *Weekly* | **0.014 (0.004, 0.023), p=0.0054** | **0.013 (0.003, 0.022), p=0.0087** | 0.018 (0.012, 0.023) | **70.2%** |
| *Weight and shape concerns* | **0.011 (0.004, 0.224), p=0.0427** | **0.097 (-0.013, 0.207), p=0.0853** | 2.255 (2.176, 2.335) | **4.3%** |
| **Disordered eating 16 years**  **(N =3,497)** |  |  |  |  |
| *Any* | **0.029 (0.005, 0.052), p=0.0213** | **0.029 (0.005, 0.052), p=0.0163** | 0.139 (0.124, 0.156) | **20.5%** |
| *Monthly* | 0.018 (-0.002, 0.038), p=0.0774 | **0.018 (-0.002, 0.038), p=0.0774** | 0.078 (0.066, 0.089) | **16.9%** |
| *Weekly* | **0.020 (0.003, 0.036), p=0.0030** | **0.018 (0.001, 0.034), p=0.0370** | 0.054 (0.043, 0.066) | **32.3%** |

**Multivariable model adjusted for**: maternal age, education, paternal profession, maternal depressive symptoms, maternal pre-pregnancy BMI, and smoking in the first pregnancy trimester.

**^a^** For disordered eating, these figures represent risk differences (we report these as proportions in the results section). For weight and shape concerns, these are mean differences.

**^b^** This potential outcome mean (POM) figure refers to the baseline risk of the outcome among the unexposed and, as such, should be interpreted as a proportion (i.e., multiplied by 100), which is how we present these figures in the Results section of manuscript. For weight and shape concerns, this represents a mean score instead.

References

1. Micali N, Daniel RM, Ploubidis GB, De Stavola BL (2018): Maternal Prepregnancy Weight Status and Adolescent Eating Disorder Behaviors: A Longitudinal Study of Risk Pathways. *Epidemiology*. 29: 579–589.

2. Harpsøe MC, Nielsen NM, Friis-Møller N, Andersson M, Wohlfahrt J, Linneberg A, *et al.* (2016): Body Mass Index and Risk of Infections Among Women in the Danish National Birth Cohort. *Am J Epidemiol*. 183: 1008–1017.

3. Bould H, Koupil I, Dalman C, DeStavola B, Lewis G, Magnusson C (2015): Parental mental illness and eating disorders in offspring. *Int J Eat Disord*. 48: 383–391.

4. Goldsmith DR, Rapaport MH, Miller BJ (2016): A meta-analysis of blood cytokine network alterations in psychiatric patients: Comparisons between schizophrenia, bipolar disorder and depression. *Mol Psychiatry*. . doi: 10.1038/mp.2016.3.

5. Larsen PS, Strandberg-Larsen K, Olsen EM, Micali N, Nybo Andersen A-M (2018): Parental characteristics in association with disordered eating in 11- to 12-year-olds: A study within the Danish National Birth Cohort. *Eur Eat Disord Rev*. . doi: 10.1002/erv.2599.

6. Zerwas S, Larsen JT, Petersen L, Thornton LM, Quaranta M, Koch SV, *et al.* (2017): Eating Disorders, Autoimmune, and Autoinflammatory Disease. *Pediatrics*. e20162089.

7. Raevuori A, Haukka J, Vaarala O, Suvisaari JM, Gissler M, Grainger M, *et al.* (2014): The Increased Risk for Autoimmune Diseases in Patients with Eating Disorders. (M. S. Horwitz, editor) *PLoS One*. 9: e104845.
